# Micro RNA 100 sensitizes luminal A breast cancer cells to paclitaxel treatment in part by targeting mTOR

**DOI:** 10.18632/oncotarget.6790

**Published:** 2015-12-29

**Authors:** Baotong Zhang, Ranran Zhao, Yuan He, Xing Fu, Liya Fu, Zhengmao Zhu, Li Fu, Jin-Tang Dong

**Affiliations:** ^1^ Department of Genetics and Cell Biology, College of Life Sciences, Nankai University, Tianjin, China; ^2^ Emory Winship Cancer Institute, Department of Hematology and Medical Oncology, Emory University School of Medicine, Atlanta, Georgia, USA; ^3^ Cancer Hospital of Tianjin Medical University, Tianjin, China

**Keywords:** miR-100, paclitaxel, mTOR, breast cancer, luminal A subtype

## Abstract

Luminal A breast cancer usually responds to hormonal therapies but does not benefit from chemotherapies, including microtubule-targeted paclitaxel. MicroRNAs could play a role in mediating this differential response. In this study, we examined the role of micro RNA 100 (miR-100) in the sensitivity of breast cancer to paclitaxel treatment. We found that while miR-100 was downregulated in both human breast cancer primary tumors and cell lines, the degree of downregulation was greater in the luminal A subtype than in other subtypes. The IC_50_ of paclitaxel was much higher in luminal A than in basal-like breast cancer cell lines. Ectopic miR-100 expression in the MCF-7 luminal A cell line enhanced the effect of paclitaxel on cell cycle arrest, multinucleation, and apoptosis, while knockdown of miR-100 in the MDA-MB-231 basal-like line compromised these effects. Similarly, overexpression of miR-100 enhanced the effects of paclitaxel on tumorigenesis in MCF-7 cells. Rapamycin-mediated inhibition of the mammalian target of rapamycin (mTOR), a target of miR-100, also sensitized MCF-7 cells to paclitaxel. Gene set enrichment analysis showed that genes that are part of the known paclitaxel-sensitive signature had a significant expression correlation with miR-100 in breast cancer samples. In addition, patients with lower levels of miR-100 expression had worse overall survival. These results suggest that miR-100 plays a causal role in determining the sensitivity of breast cancers to paclitaxel treatment.

## INTRODUCTION

Breast cancer is a major disease for which more effective and efficient therapies need to be developed. Molecular expression profiling, in combination with the expression status of estrogen receptor (ER), progesterone receptor (PR) and human epidermal growth factor receptor 2 (HER2) [1-3], has led to the classification of breast cancers into four subtypes [4, 5]: luminal A (ER+, PR+ and HER2-), luminal B (ER+, PR+ and HER2+), HER2 positive (ER-, PR- and HER2+), and basal-like (ER-, PR- and HER2-, or triple negative) [3]. Each subtype has distinct biological behavior, prognosis, and usually different responses to different chemotherapies, yet the key molecular mechanisms underlying the responses of breast cancer subtypes to specific drugs largely remain to be clarified [2, 5]. For example, while luminal A breast cancers generally have a good prognosis and respond well to hormonal therapies, they do not appear to benefit from the addition of the microtubule-targeted chemotherapy drug paclitaxel [2, 6-8]. The underlying mechanisms for this are still unclear, although a gene signature has been suggested based on expression profiling of breast cancers that respond or do not respond to paclitaxel treatment [9].

MicroRNAs are a group of small non-coding RNAs that function as gene regulators in a variety of biological processes [10]. For example, miR-100 regulates apoptosis, autophagy, cell growth and survival, migration, stem cell self-renewal and drug sensitivity by targeting different molecules such as mammalian target of rapamycin (mTOR), PLK1 and SMARCA5 [11-13]. MiR-100 appears to play a role in cancer development. For example, miR-100 is commonly downregulated in various types of human tumors [14-20], and lower levels of miR-100 expression correlate with poorer prognosis in patients with several types of malignancies such as esophageal squamous cell carcinoma, colorectal cancer, hepatocellular carcinoma and bladder cancer [14-17, 21]. In breast cancer, miR-100 also appears to play a role, as miR-100 induces epithelial-mesenchymal transition but suppresses tumorigenesis, migration and invasion [11]. The tumor suppressor function of miR-100 appears to involve the proliferation and survival-promoting oncogene insulin-like growth factor (IGF) 2 [22]. However, the role of miR-100 in breast cancer remains to be clarified. For example, while miR-100 regulates β-tubulin isotypes in MCF-7 breast cancer cells [23], and tubulin alteration is a known mechanism for breast cancer resistance to the microtubule-targeted drug paclitaxel [24], luminal A breast cancers are less responsive to paclitaxel treatment, and the role of miR-100 in paclitaxel response is unknown. In addition, while miR-100 inhibits the self-renewal of breast cancer stem-like cells (BrCSCs) and sensitizes basal-like breast cancer stem cells to hormonal therapy by promoting cell differentiation [25, 26], whether miR-100 has different effects on different subtypes of breast cancer remains unknown.

In this study, we evaluated the role of miR-100 in different subtypes of breast cancer, with a focus on paclitaxel response. Considering that mTOR, an important player in PI3K oncogenic signaling, has been identified as a target molecule of miR-100 [13, 27], and combined use of rapamycin and paclitaxel improves the chemotherapeutic effect of either in breast cancer [28], we also tested whether miR-100 affects the response of breast cancer cells to paclitaxel by targeting mTOR. Expression evaluation, functional and molecular tests, and bioinformatic and survival analyses suggest that miR-100 plays a role in breast cancer development by promoting paclitaxel sensitivity in part by targeting mTOR.

## RESULTS

### MiR-100 is downregulated in human breast cancer, particularly the luminal A subtype

**Figure 1:**
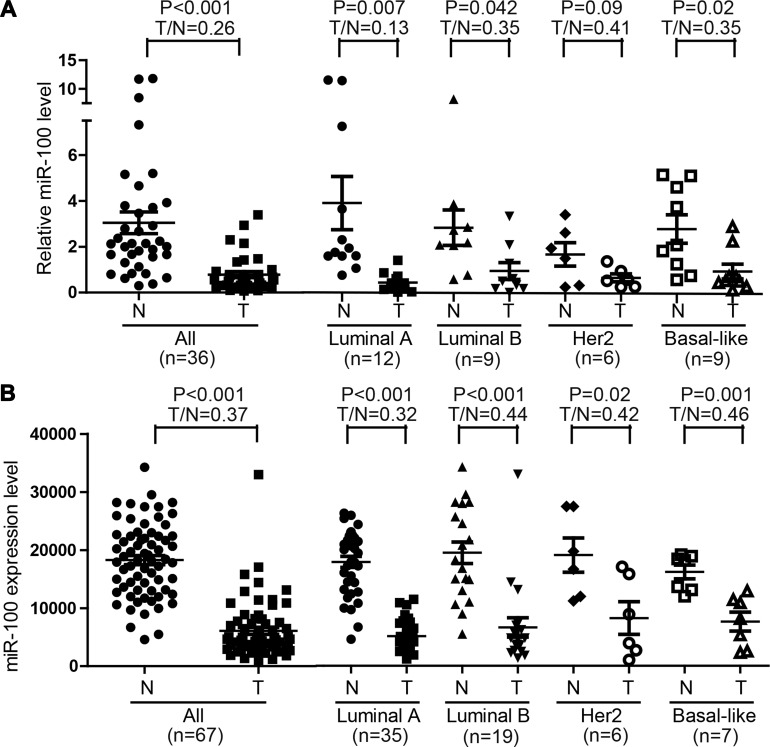
Downregulation of miR-100 in breast cancer **A.** Detection of miR-100 expression by real-time RT-PCR in 36 breast cancer specimens and their matched adjacent normal tissues. **B.** Expression of miRNA-100, as determined by RNA sequencing, in 67 breast cancer samples and their matched normal tissues in the TCGA database. The T/N ratio of miR-100 expression in luminal A tumors was significantly lower when compared to the remaining samples in A (*P* < 0.05).

To clarify the function of miR-100 in breast cancer, we first evaluated the expression of miR-100 by real-time PCR in 36 breast cancer specimens, with each cancer's adjacent normal breast tissue as the control. In addition to confirming a significant downregulation of miR-100 in breast cancer tissues (Figure 1A), we noticed that miR-100 downregulation occurred in each of the four subtypes of breast cancers, luminal A, luminal B, HER2 and basal-like (Figure 1A). Interestingly, the ratio of miR-100 level in a tumor to that in matched normal control, which indicates the extent of miR-100 downregulation, was significantly smaller in luminal A tumors than in other tumors (Figure 1A), indicating that miR-100 downregulation was more extensive in the luminal A subtype of breast cancer.

Similar results were obtained when miR-100 expression, as determined by RNA-Seq, was analyzed in the group of breast cancer samples that had matched normal tissues with complete ER, PR and HER2 status in the TCGA database (Table S1). Again, miR-100 was downregulated in these breast cancers, and the downregulation was more pronounced in luminal A breast cancers than in other subtypes of tumors (Figure 1B).

### MiR-100 sensitizes breast cancer cells to paclitaxel inhibition of cell proliferation and survival

**Figure 2:**
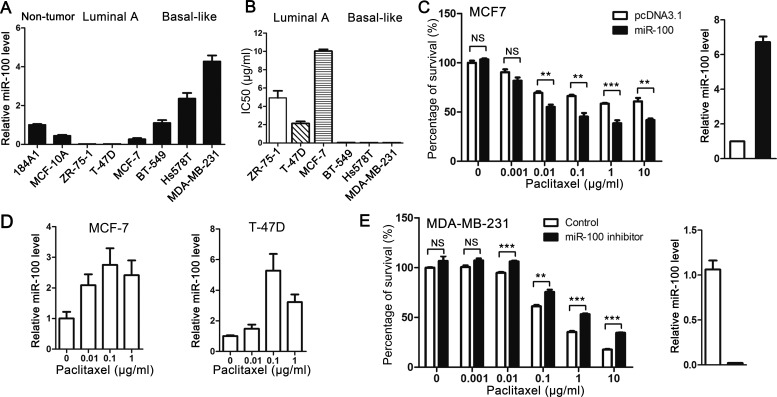
Expression of miR-100 sensitizes breast cancer cells to the cytotoxic effect of paclitaxel *in vitro* **A.** Expression levels of miR-100, as determined by real-time PCR, in noncancerous breast epithelial cell lines (184A1 and MCF10A) and breast cancer cell lines of luminal A subtype (ZR-75-1, T-47D and MCF-7) and basal-like subtype (BT-549, Hs 578T and MDA-MB-231). **B.** Luminal A breast cancer cell lines are more resistant to paclitaxel than basal-like lines in culture, as indicated by their IC50 values. **C.** Ectopic expression of miR-100 sensitizes the MCF-7 luminal A breast cancer cell line to paclitaxel. Ectopic expression of miR-100 was confirmed by real-time PCR (panel at right). **D.** Paclitaxel treatment induces miR-100 expression in luminal A breast cancer cell lines MCF-7 and T-47D, as detected by real-time PCR. **E.** Inhibition of miR-100 desensitizes the MDA-MB-231 basal-like breast cancer cell line to paclitaxel. Inhibition of miR100 expression was confirmed by real-time PCR (panel at right). NS, not significant; *, *P* < 0.05; **, *P* < 0.01; ***, *P* < 0.001.

Compared to other subtypes of breast cancer, luminal A cancers are responsive to hormonal therapy but more resistant to chemotherapies including paclitaxel treatment [2, 6-8]. Considering the more severe miR-100 downregulation in luminal A cancers (Figure 1), it is possible that miR-100 is functionally involved in breast cancer sensitivity to paclitaxel's cytotoxic effect. To test this possibility, we first evaluated miR-100 expression by real-time PCR in 3 luminal A (ZR-75-1, T-47D and MCF-7) and 3 basal-like (BT-549, Hs 578T and MDA-MB-231) breast cancer cell lines [29, 30], with immortalized noncancerous breast epithelial cell lines 184A1 and MCF10A as references. Compared to the noncancerous lines and 3 basal-like lines, the 3 luminal A cell lines expressed much less miR-100 (Figure 2A), consistent with the pattern of miR-100 expression in the two subtypes seen in human breast cancer specimens (Figure 1).

We then determined IC_50_ values of paclitaxel in the 6 breast cancer cell lines. The IC_50_ values of paclitaxel were much higher in the 3 luminal A lines (ranging from 2 to 10 μg/ml), all of which had lower levels of miR-100 expression, than in the 3 basal-like breast cancer cell lines (less than 0.05 μg/ml) (Figure 2B). IC_50_ values between the 2 groups of breast cancer cell lines were significantly correlated with miR-100 expression levels (P < 0.001), supporting the role of miR-100 in the sensitivity of breast cancer cells to paclitaxel treatment.

To determine whether miR-100 plays a causal role in paclitaxel response, we increased miR-100 expression in MCF-7 cells to a level similar to that in basal-like breast cancer cell lines, as determined by real-time PCR (Figure 2C, panel at right), and then measured the effect of paclitaxel on cell proliferation and survival using the CCK8 assay. While restoration of miR-100 expression did not change cell proliferation or survival (Figure 2C, bars at far left), it significantly enhanced the effect of paclitaxel even at the low concentration of 1 ng/ml (Figure 2C, panel at left), with IC_50_ decreasing from 9.6 μg/ml (9.56 ± 1.8) to 0.05 μg/ml (0.05 ± 0.02). In addition, paclitaxel induced miR-100 expression in two luminal A breast cancer cell lines, MCF-7 and T-47D (Figure 2D), suggesting that miR-100 can serve as an effector in paclitaxel-induced cellular responses.

In the MDA-MB-231 basal-like breast cancer cell line, which expressed a much higher miR-100 level than MCF-7 cells (Figure 1) and was much more sensitive to paclitaxel (Figure 2B), knockdown of miR-100 expression did not affect cell proliferation or survival with no or lower concentrations of paclitaxel (0-0.01 μg/ml) but significantly compromised the effect of higher concentrations of paclitaxel (0.1-10 μg/ml) (Figure 2E). These results indicate that miR-100 plays a causal role in breast cancer cell sensitivity to the inhibitory effect of paclitaxel on cell proliferation and survival.

### Expression of miR-100 sensitizes MCF-7 tumors to paclitaxel treatment

**Figure 3:**
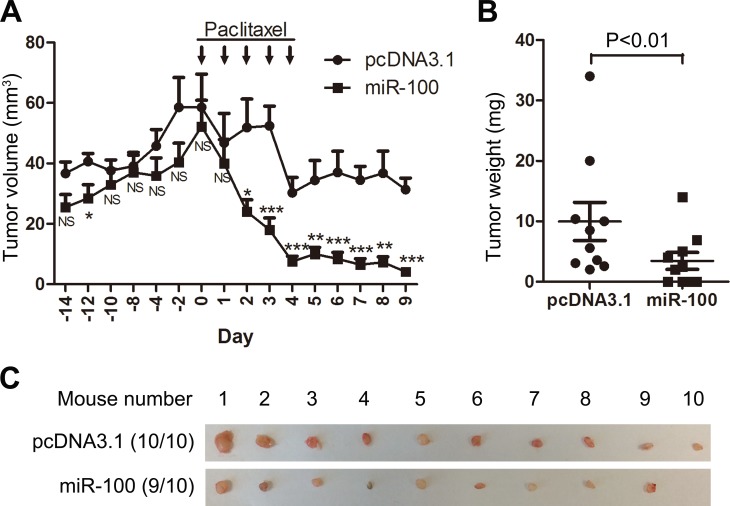
Ectopic expression of miR-100 sensitizes MCF-7 luminal breast cancer to the therapeutic effects of paclitaxel in nude mice MCF-7 cells with miR-100 expression, along with control cells (pcDNA3.1), were injected into both sides of nude mice to initiate tumorigenesis, with paclitaxel treatment administered 14 days post inoculation. **A.** Tumor volumes at different time points, **B.** tumor weights and **C.** tumor images at excision. Results for each group were from 10 mice. NS, not significant; *, *P* < 0.05; **, *P* < 0.01; ***, *P* < 0.001.

We also performed tumorigenesis assays to determine whether miR-100 expression improves the therapeutic effect of paclitaxel on breast cancer. MCF-7 breast cancer cells were transfected with miR-100 expression plasmid and control vector, and cells stably expressing exogenous miR-100 or control vector were injected subcutaneously into nude mice, with or without paclitaxel treatment. During the 3 weeks of tumor growth before paclitaxel administration, miR-100 expression appeared to reduce tumor volumes but the reduction was not statistically significant (Figure 3A). After paclitaxel treatment was started, tumor growth was significantly suppressed. This suppression was much more profound in tumors expressing ectopic miR-100, as indicated by significantly reduced tumor volumes from day 2 after paclitaxel administration (Figure 3A), tumor weights at excision (Figure 3B), and tumor images (Figure 3C). These results indicate that restoration of miR-100 expression sensitizes MCF-7 breast cancer cells to paclitaxel treatment in a xenograft model.

### MiR-100 enhances the effect of paclitaxel on cell cycle arrest, multinucleation and apoptosis

**Figure 4:**
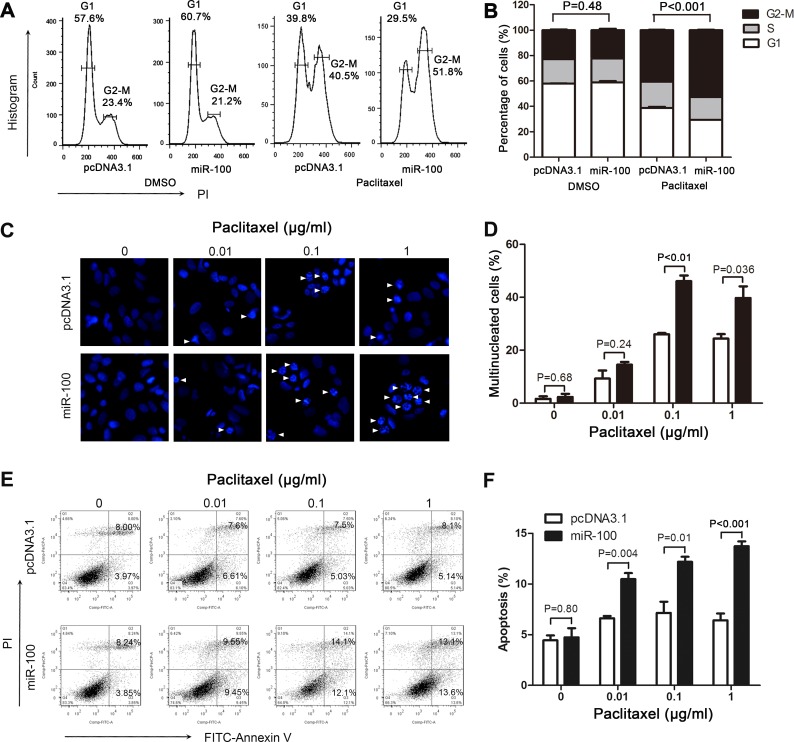
MiR-100 sensitizes MCF-7 cells to paclitaxel by promoting paclitaxel-induced cell cycle arrest, multinucleation and apoptosis MCF-7 cells with or without miR-100 expression (miR-100 *vs*. pcDNA3.1) were treated with paclitaxel for 48 hours and then subjected to flow cytometry analysis for cell cycle arrest **A.**, **B.**, immunofluorescence staining for multinucleation **C.**, **D.**, and flow cytometry analysis for apoptosis **E.**, **F.**. More cells with miR-100 overexpression and paclitaxel treatment (0.01 μg/ml) were arrested at the G2-M phase **A.**, **B.**, had multinucleation **C.**, **D.**, and underwent early or late apoptosis **E.**, **F.**. Arrows in panel C point to cells with multinucleation.

Paclitaxel treatment induces cell cycle arrest, multinucleation and apoptosis [31, 32]. We examined these cellular processes in paclitaxel-treated MCF-7 cells with or without miR-100 overexpression to determine their involvement in miR-100-mediated sensitization to paclitaxel. While miR-100 overexpression did not itself alter cell cycle progression, it significantly enhanced the induction of cell cycle arrest at the G2/M phase by paclitaxel treatment (Figure 4A and 4B). Similarly, while miR-100 overexpression did not induce detectable multinucleation and apoptosis, it significantly enhanced the induction of both multinucleation and apoptosis observed following paclitaxel treatment (Figure 4C-4F). These findings indicate a role of miR-100 in paclitaxel-induced cell cycle arrest, multinucleation and apoptosis.

### One target of miR-100, mTOR, is involved in paclitaxel sensitivity

**Figure 5:**
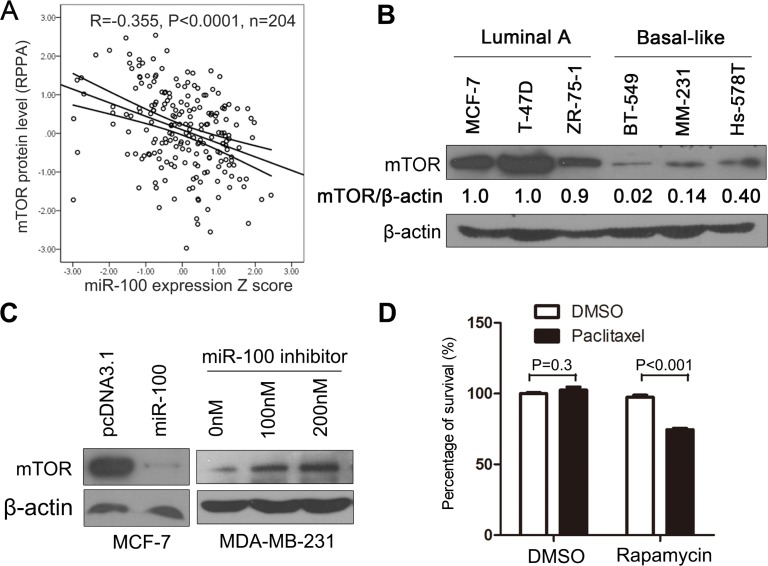
mTOR plays a role in miR-100-mediated sensitization to paclitaxel treatment **A.** Expression level of miR-100 inversely correlates with that of mTOR protein in breast cancer samples, as determined by Pearson correlation analysis using data from the TCGA database (Table S4). **B**. Expression level of mTOR protein is higher in luminal A breast cancer cell lines than basal-like lines, as detected by Western blotting. **C.** Overexpression of miR-100 inhibits, while inhibition of miR-100 promotes, mTOR expression in MCF-7 and MDA-MB-231 breast cancer cell lines respectively, as detected by Western blotting. β-actin in panels A and B served as the loading control. **D.** Inhibition of mTOR by rapamycin treatment sensitizes MCF-7 breast cancer cells to cell death induced by a low concentration of paclitaxel (0.001 μg/ml), as determined by the CCK-8 assay.

Micro-RNAs target gene transcripts, often by inducing their degradation or inhibiting their protein translation, to regulate biological processes. We therefore performed a series of analyses to identify the molecular target(s) of miR-100 involved in breast cancer sensitivity to paclitaxel treatment. Based on previous publications, we first summarized the major cellular processes and underlying molecules that determine breast cancer sensitivity or resistance to paclitaxel treatment (Table S2). We then collected all molecules that had been identified as target molecules of miR-100 (Table S3). By comparing molecules involved in both groups, we found that mTOR was the only known miR-100 target that has also been shown to affect breast cancer sensitivity to paclitaxel. To evaluate whether mTOR is a target molecule of miR-100 in breast cancer, we collected breast cancer samples in the TCGA database that had expression information for both miR-100 and mTOR protein (Table S4), and looked for correlations between their expression levels. A significant inverse correlation was found between miR-100 and mTOR in invasive breast cancers (Figure 5A), implicating mTOR as a target of miR-100 in breast cancer.

To experimentally test whether mTOR plays a role in miR-100-mediated sensitivity to paclitaxel, we first measured mTOR protein expression in the 6 luminal A and basal-like breast cancer cell lines used for expression and functional analyses of miR-100. The expression of mTOR was significantly higher in the 3 luminal A cell lines than in the basal-like lines (P < 0.01) (Figure 5B). The pattern was the inverse of that seen for miR-100 expression (Figure 2A). Additional experiments further confirmed that mTOR is indeed a target of miR-100 in breast cancer cells. When miR-100 was ectopically expressed in MCF-7 cells, the mTOR protein level was dramatically decreased (Figure 5C, panel at left). Consistently, when miR-100 expression was inhibited in MDA-MB-231 cells, the mTOR protein level was increased (Figure 5C, panel at right).

To functionally evaluate whether mTOR plays a role in miR-100-mediated paclitaxel sensitization, we treated MCF-7 breast cancer cells with a concentration of paclitaxel (1 ng/ml) that was too low to affect cell proliferation and survival. Under these conditions, inhibition of mTOR by rapamycin, also at a concentration that was too low to suppress cell proliferation and survival, significantly enhanced the inhibitory effect of paclitaxel on cell proliferation and survival (Figure 5D). This result confirms that mTOR plays a role in miR-100-mediated sensitization of breast cancer cells to paclitaxel.

### Implication of miR-100 in the treatment of human breast cancer with paclitaxel

**Figure 6:**
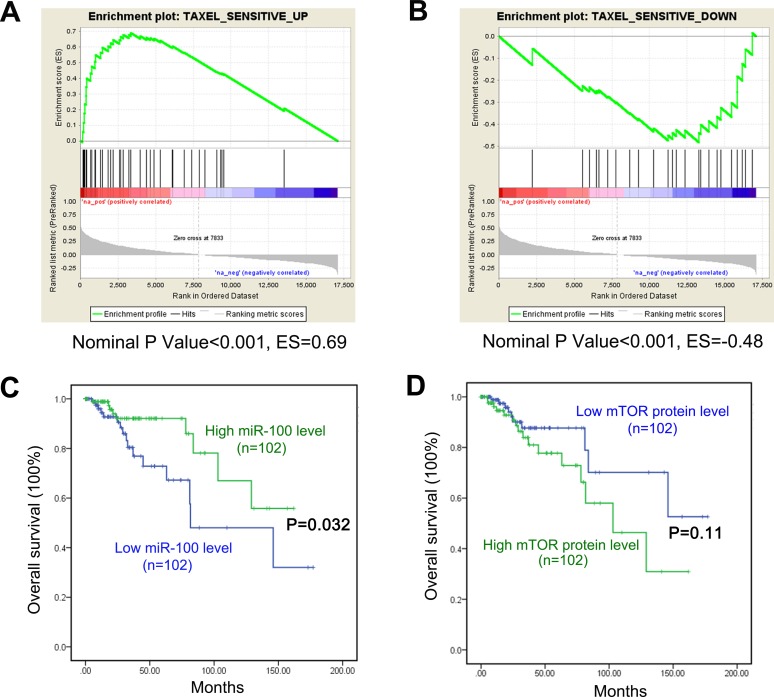
Implication of miR-100 in breast cancer response to paclitaxel treatment **A.** Genes upregulated in breast cancers that responded to paclitaxel treatment, when compared to those that did not respond, were significantly enriched among genes whose expression levels positively correlated with miR-100 expression, as determined by Gene Set Enrichment Analysis (GSEA). **B.** Genes downregulated in paclitaxel-responsive tumors were significantly enriched among genes whose expression levels negatively correlated with miR-100 expression in breast cancer, also determined by GSEA. For A and B, green curves indicate enrichment scores. The heat map lists genes in the order of strong positive correlation (red) to strong negative correlation with miR-100 expression (green). The lower gray panels show the Pearson's R score for each ranked gene in the heat map. Vertical lines above the heat map indicate the genes constituting the paclitaxel-sensitive signature of breast cancer. ES, Enrichment score. **C.** and **D.** Patients with lower miR-100 levels had worse overall survival than those with higher miR-100 expression, as analyzed using data from the TCGA database **C.**. In the same group of patients, those with higher mTOR protein expression had a trend toward worse survival but the difference was not statistically significant **D.**

To explore whether patients with breast cancers with higher miR-100 expression levels benefit more from paclitaxel treatment, we analyzed whether the expression of miR-100 correlative genes is associated with the expression of genes in the paclitaxel-sensitive signature by using gene set enrichment analysis (GSEA). The paclitaxel-sensitive gene signature was established in a previous study [9], and consists of genes that were either upregulated or downregulated in breast cancers from patients with a positive response to paclitaxel treatment when compared to those from unresponsive patients. We used the cBioPortal program and the TCGA database to determine the expression change for every gene between cancers and their matched normal tissues, and then calculated the correlation of these changes to those of miR-100 [33]. All genes were then ranked based on their correlation scores [33]. We found that genes upregulated in the paclitaxel-sensitive signature were significantly enriched among genes that positively correlated with miR-100 expression (Figure 6A), while genes downregulated in the paclitaxel sensitive signature were enriched among genes whose expression was inversely correlated with miR-100 expression (Figure 6B). When the breast cancer samples were divided according to their subtypes for the GSEA, the correlation was still significant in luminal A and B subtypes but not in the basal-like and HER2 tumor subtypes (data not shown). These results suggest that breast cancer patients with higher tumor miR-100 expression, particularly those with luminal A and B subtypes, benefit more from paclitaxel treatment.

We also performed survival analyses for the group of breast cancer patients from the TCGA database (Table S4). Patients with lower miR-100 expression in their cancers had worse overall survival (Figure 6C). For mTOR, patients with higher tumor protein levels had a trend of worse survival but the difference did not reach statistical significance (Figure 6D).

## DISCUSSION

### MiR-100 causally affects paclitaxel sensitivity in breast cancer

Luminal A breast cancers are more resistant to chemotherapies including paclitaxel treatment [2, 6-8], and the greater degree of downregulation of miR-100 in luminal A breast cancers (Figure 1) suggests a role for miR-100 in determining breast cancer sensitivity to paclitaxel treatment. Our study provides several lines of evidence indicating that this is indeed the case. First, when breast cancer cell lines were classified into different subtypes based on the expression status of ER, PR and HER2 [29, 30], luminal A lines expressed much lower levels of miR-100 than basal-like lines (Figure 2), which was consistent with our finding in breast cancer tissues (Figure 1). Interestingly, the IC_50_ of paclitaxel was much greater in the luminal A lines than in the basal-like lines (Figure 2B), providing functional support for the role of miR-100 in paclitaxel sensitivity. Second, in the MCF-7 luminal A breast cancer cell line, which expressed a lower level of miR-100 (Figure 2A), ectopic expression of miR-100 sensitized cells not only to paclitaxel-induced cell cycle arrest and apoptosis *in vitro* (Figures 2, 4) but also to tumor suppression in nude mice (Figure 3), providing direct functional evidence. Consistently, in MDA-MB-231 basal-like cells, which expressed a higher level of miR-100 and were very sensitive to paclitaxel (Figure 2A, 2B), inhibition of miR-100 expression desensitized cells to paclitaxel-induced cell proliferation and survival (Figure 2E). Third, of the published gene expression signature that predicts a positive response of patients to paclitaxel, the upregulated genes positively correlated, and the downregulated genes negatively correlated, with miR-100 expression in human breast cancer (Figure 6). In addition, paclitaxel treatment increased miR-100 expression level in the luminal A breast cancer cell lines we tested (Figure 2D). We therefore conclude that miR-100 affects the therapeutic response of breast cancer to paclitaxel, and that patients with higher levels of miR-100 expression benefit more from paclitaxel treatment.

The role of miR-100 in paclitaxel sensitivity does not seem specific to luminal A breast cancer, because the downregulation of miR-100 was also frequent and significant in other subtypes of breast cancer (Figure 1), and knockdown of miR-100 in MDA-MB-231 basal-like breast cancer cells desensitized their response to paclitaxel. It is unknown why and how luminal A breast cancers have a greater degree of miR-100 downregulation compared to other subtypes.

### MiR-100 sensitizes breast cancer cells to paclitaxel by targeting mTOR and other mechanisms

As a miRNA, miR-100 targets a number of genes for translational regulation. Previous studies identified mTOR as a direct target of miR-100 in the promotion of apoptosis [34] and autophagy [35] and in the inhibition of cell proliferation [36] (Table S3). Our expression analysis in human breast cancer tissues and cell lines, including cell lines where miR-100 expression was manipulated, further confirmed mTOR as a target of miR-100 (Figure 5A, 5B, 5C). Inhibition of mTOR by rapamycin enhances paclitaxel-induced cell death in MCF-7 cells [28], and our results showed that even a very low concentration of paclitaxel enhanced the effect of rapamycin on cell death (Figure 5D). Therefore, targeting mTOR appears to be an important mechanism by which miR-100 sensitizes breast cancer cells to paclitaxel.

Other targets of miR-100 may also contribute to its effects on breast cancer cell sensitivity to paclitaxel. Paclitaxel causes exit from mitosis into a G1-like multinucleated state, and multinucleated cells undergo apoptosis because of DNA damage [31]. Different doses and treatment times of paclitaxel also have different effects on these processes, as longer treatments and higher doses induce a shift from cell cycle arrest to multinucleation and further to apoptosis [31, 32]. Our results showed that miR-100 overexpression enhanced the effect of paclitaxel on cell cycle arrest, multinucleation and apoptosis (Figure 4). In addition, among the large number of miR-100 targets that have been identified in published studies (Table S3), PLK1 kinase has also been shown in a number of studies to function in cell proliferation and apoptosis. It is thus possible that, like mTOR, PLK1 may also mediate the effect of miR-100 on breast cancer sensitivity to paclitaxel, though this remains to be determined.

In several breast cancer cell lines including the MCF-7 luminal A line, miR-100 overexpression decreases the number of ALDH positive cells, which represent a stem-like subpopulation [25]. Consistently, miR-100 overexpression in MCF-7 cells inhibited mammosphere formation (data not shown), which indicates the stem-like feature of cancer cells. Furthermore, miR-100 overexpression in basal-like cells not only inhibits the maintenance and expansion of breast cancer stem-like cells (BrCSCs), it also promotes their differentiation or conversion from a basal-like phenotype to a luminal phenotype [26]. In addition, GSEA showed that the enrichment of genes conferring paclitaxel sensitivity in miR-100-corresponding genes was more profound in the luminal A and B subtypes than in basal-like and HER2 subtypes, and the former two are more differentiated than the latter two. Therefore, while the mechanism by which luminal A breast cancers appear to be more affected by miR-100 is unknown, one possibility is that miR-100 plays a more crucial role in the luminal differentiation of breast epithelial cells, and more severe downregulation of miR-100 is important for the formation of luminal breast cancers.

### MiR-100 plays a suppressor role in breast cancer

Previous studies suggest that miR-100 plays a tumor suppressor role in breast cancer, as it suppresses the migration, invasion and tumorigenesis of breast cancer cells, inhibits the self-renewal of BrCSCs, and promotes the differentiation of BrCSCs[11, 22, 25, 26]. In this study, we provided another line of evidence for a tumor suppressor role of miR-100 in breast cancer, as we found that miR-100 was frequently downregulated in human breast cancer, and the extent of downregulation was greater in the luminal A subtype of tumors than in other subtypes (Figure 1). In addition, miR-100 downregulation in breast cancer was associated with worse overall survival in breast cancer patients (Figure 6). Ectopic expression of miR-100 sensitized, while inhibition of miR-100 expression desensitized, breast cancer cells to the therapeutic effect of paclitaxel both *in vitro* and in xenograft tumorigenesis (Figures 2 and 3). The association of lower miR-100 expression with worse patient survival has also been reported in other types of cancers, including esophageal squamous cell carcinoma [14], colorectal cancer [15], hepatocellular carcinoma [16], bladder cancer [17], ovarian cancer [21] and non-small cell lung cancer [20]. In addition, detection of miR-100 in the serum of cancer patients appears to have diagnostic and prognostic value as a potential biomarker [19, 37-40]. Our findings in this study suggest that miR-100 may also have therapeutic value. For example, enhancement or restoration of miR-100 expression in cancer cells, particularly in luminal A breast cancer, may sensitize these cells to paclitaxel treatment.

In summary, we found that miR-100 expression was significantly downregulated in breast cancer, and the downregulation was more extensive in luminal A breast cancers and was associated with worse patient survival. Ectopic expression of miR-100 sensitized, while inhibition of miR-100 expression desensitized, breast cancer cells to the effect of paclitaxel on cell cycle arrest, multinucleation, apoptosis and tumorigenesis. Expression of genes that are part of a known signature of paclitaxel sensitivity in breast cancer significantly correlated with miR-100 expression. Mechanistically, targeting mTOR appeared to mediate miR-100's function in sensitizing breast cancer cells to paclitaxel, but other mechanisms also seem to be involved, including targeting other molecules such as PLK1. These findings suggest that miR-100 plays a role in breast cancer development, and its detection and expression have diagnostic, prognostic and therapeutic value in the detection and treatment of breast cancer.

## MATERIALS AND METHODS

### Plasmid construction

The pcDNA3.1-miR-100 expression vector was constructed by PCR amplification of the genomic DNA containing pre-miR-100 with primers 5′-TCCGGAATTCGTGGAAACCAAGGGAAGC-3′ and 5′-CTAGTCTAGATTGAGGGCCAGCCTATTA-3′, digestion with *Eco*RI and *Xba*I, and subsequent cloning into the pcDNA3.1 plasmid (Invitrogen, Carlsbad, CA).

### Cell lines, primary tumor specimens and special reagents

Breast cancer cell lines used in this study included T-47D, MCF-7, ZR-75-1, BT-549, MDA-MB-231 and Hs578T, all of which were purchased from the American Type Culture Collection (ATCC, Manassas, VA) and propagated as previously described [41]. STR analyses have been used for cell line authentication within 6 months. MCF-7 cells with ectopic expression of miR-100 were obtained by transfecting pcDNA3.1-miR-100 plasmids into cells, along with the vector control, and selection with G418-containing medium (800 μg/ml) for at least 2 weeks after transfection.

Breast cancer specimens and matched adjacent noncancerous breast tissues from 36 patients were obtained from the Department of Breast Cancer Pathology and Research Laboratory, Cancer Hospital of Tianjin Medical University, Tianjin, China. Samples were free of hemorrhagic and necrotic areas, snap frozen within half an hour after surgery in liquid nitrogen, and stored in a −80°C freezer until use for RNA isolation.

### RT-PCR and real-time PCR

Total RNA was isolated from cells using Trizol reagent (Invitrogen), and the cDNA for miRNA was synthesized with the stem-loop primer 5′ - GTCGTATCCGAGGTATTCGCACTGGATACGACC ACAAG - 3′) using the RT kit from Promega (Fitchburg, WI). Real-time PCR with SYBR green (Takara, Dalian, China) was performed with the Realplex Real-time PCR Detection System (Eppendorf, Beijing, China) to detect the expression of miR-100, with the 5′-GTGCAGGGTCCGAGGT-3′ (forward) and 5′-CCCGTAGATCCGAACTTG-3′ (reverse) primer sequences. U6 was used as an internal control with 5′-CTCGCTTCGGCAGCACA-3′ and 5′-AACGCTTCACGAATTTGCGT-3′ PCR primers.

### Western blotting

Western blotting was performed as described in our previous publication [42]. Rabbit polyclonal mTOR antibody was from Cell Signaling (catalogue #2972, Beverly, MA) and was used at a dilution of 1:1000. Mouse β-actin monoclonal antibody was from Sigma (Beijing, China) and was used at 1:2000 dilution. Intensities of protein bands were measured using NIH ImageJ software [43, 44].

### Cytotoxicity assay

Cells were seeded in 96-well plates at 5000 cells per well, allowed to adhere overnight, and incubated in medium containing paclitaxel at various concentrations for 48 hours. Total cell numbers were determined using the Cell Counting Kit-8 (DojinDo, Beijing, China). Briefly, cells were incubated for 2 hours at 37°C after the addition of 10 μl CCK-8 solution per well, and the optical density for each well was measured using an EMax Precision microplate reader (Molecular Devices, Shanghai, China) at a 450 nm wavelength. Each treatment was performed at least in triplicate.

### Cell cycle analysis

MCF7 cells with or without miR-100 overexpression were exposed to paclitaxel for 48 hours as described above, and then collected and fixed with 70% ethanol for at least 24 hours. After washing, cells were resuspended in PBS, incubated with propidium iodide (10 mg/ml, Sigma) and RNase A (20 mg/ml) for 30 min in the dark, and subjected to flow cytometry analysis with a BD FACSCalibur flow cytometer (Franklin Lakes, NJ).

### Apoptosis assay

Apoptosis was measured by staining the cells with Annexin V-FITC and PI. After incubation with paclitaxel for 48 hours, cells were collected, washed with cold PBS, resuspended in 100 μl of 1 x Annexin V binding buffer, stained with Annexin V and PI (BD Pharmingen) by adding 5 μl of each to each tube and incubating for 15 min at room temperature in the dark, followed by flow cytometry analysis. Data were analyzed using FlowJo 7.6 software.

### Multinucleation analysis

Cells were fixed in 4% paraformaldehyde for 20 min, permeabilized with 0.5% (vol/vol) Triton X-100 for 10 min, blocked with 2% BSA for 2 hours at room temperature, and stained with 4′,6-diamidino-2-phenylindole (DAPI) (1:1000) for 15 min. Fluorescence images were taken at room temperature with a Leica DM4000B microscope (Wetzlar, Germany) equipped with a DFC490 charge coupled device camera.

### Tumorigenesis assay in nude mice

MCF-7 cells with and without miR-100 expression were resuspended in a solution of Matrigel (BD USA) and PBS (equal volume), and then inoculated subcutaneously into the flanks of 8 week old female BALB/c nude mice (Charles River, San Diego, CA) at 3 × 10^6^ cells per site. Pellets of 17β-estradiol (0.72 mg, 60-day release; Innovative Research of America, Sarasota, FL) were implanted subcutaneously in the shoulder region of each mouse one day before cell inoculation. Three weeks after cell inoculation, mice were given daily intraperitoneal injections of paclitaxel (20 mg/kg b.w) or the solvent DMSO for 5 consecutive days according to a previously described procedure [45]. Tumor volumes were measured every other day before paclitaxel administration and daily after the initiation of paclitaxel treatment. At the end of the tumorigenesis assay, tumors were surgically isolated from mice and their weights measured.

### Bioinformatic analyses

Publically available databases were used for bioinformatic analyses. MiR-100 expression in breast cancer tissues and their adjacent normal tissues, as determined by the Illumina HiSeq and Illumina Genome Analyzer and indicated by normalized read counts, were retrieved from the Cancer Genome Atlas (TCGA) database (http://cancergenome.nih.gov) using the Synapse platform (http://synapse.org) (syn1461151) [11]. Surrogate subtypes of breast cancer in the TCGA database, as defined by the IHC status of ER, PR and HER2 in patient pathological reports, were retrieved from the Cancer Digital Slide Archive [46]. The cBioPortal program [33] was applied to the TCGA database to identify invasive breast carcinomas that had information on the expression levels of both miR-100 and mTOR protein. The latter was based on the reverse phase protein array (RPPA).

To identify genes whose expression changes corresponded to that of miR-100 in breast cancer, we used the cBioPortal program to rank the expression change of each gene between a cancer and its normal control compared with miR-100's expression change in the same tumor and normal samples. For paclitaxel-responsive genes, we used a published microarray dataset [9], in which paclitaxel-responsive genes were identified by comparing pre-treatment breast cancer biopsies from patients who had a pathologic complete response (pCR) to paclitaxel treatment to those from patients who had no pCR (NR). Some paclitaxel-responsive genes were upregulated while some were downregulated in biopsies of patients with pCR to paclitaxel treatment.

As previously described [47], we used the Gene Set Enrichment Analysis (GSEA) software to calculate enrichment scores for paclitaxel-responsive genes and their expression correlation with miR-100 expression. GSEA calculates the enrichment score by walking down the ranked list of genes, increasing a running-sum statistic when a gene is in the gene set and decreasing the running-sum when a gene is not in the set. The magnitude of the increment depends on the correlation of the gene with a phenotype (paclitaxel response). ES is the maximum deviation from zero encountered in walking the list, reflecting the degree to which a gene set is overrepresented at the top or bottom of a ranked list of genes (e.g., miR-100-responsive genes). A positive ES indicates gene set enrichment at the top of the ranked list, and a negative ES indicates gene set enrichment at the bottom of the ranked list.

### Statistical analysis

All experimental readings were expressed as mean ± standard errors. Differences between two groups were determined by using the unpaired Student *t*-test, and p-values less than 0.05 were considered as statistically significant. In addition, the Spearman correlation analysis was applied to the mean of miR-100 expression levels and that of paclitaxel IC50 in luminal A and basal-like breast cancer cell lines to determine the correlation between miR-100 expression, IC50 and subtypes of breast cancer.

## SUPPLEMENTARY TABLES








