# Supplementary material for: Micro RNA 100 sensitizes luminal A breast cancer cells to paclitaxel treatment in part by targeting mTOR
Source: Oncotarget. 2015 Dec 29;7(5):5702–14. doi: 10.18632/oncotarget.6790 (PMC4868715; doi:10.18632/oncotarget.6790)
Supplement: Supplementary file 2 [file oncotarget-07-5702-s002.pdf]

Table S2. Key cellular processes and related proteins that mediate the resistance of breast cancer to paclitaxel treatment.

Cellular processes	Key proteins
Apoptosis	Bcl2 [1,2], mTOR [3], ErbB2 [4], JNK [5], Cyr61/NF-κB/XIAP [6], STAT3 [7]
Mitosis	BRCA1 [8,9], FBW7-MCL1 [10], PARKIN [11], CLIP-170 [12], SEPT10 [13], ErbB2 [14]
Multidrug resistance	P-gp [15-17], ERα [18].
Cancer stemness	AKT [19], Twist-Bcl2 [20]
Autophagy	mTOR [21]

References:

1. Zhou T, Duan J, Wang Y, Chen X, Zhou G, Wang R, Fu L, Xu F. Fluoxetine synergys with anticancer drugs to overcome multidrug resistance in breast cancer cells. *Tumour Biol* 2012;**33**:1299-306.
2. Hawthorne VS, Huang WC, Neal CL, Tseng LM, Hung MC, Yu D. ErbB2-mediated Src and signal transducer and activator of transcription 3 activation leads to transcriptional up-regulation of p21Cip1 and chemoresistance in breast cancer cells. *Mol Cancer Res* 2009;**7**:592-600.
3. Dong J, Peng J, Zhang H, Mondesire WH, Jian W, Mills GB, Hung MC, Meric-Bernstam F. Role of glycogen synthase kinase 3beta in rapamycin-mediated cell cycle regulation and chemosensitivity. *Cancer Res* 2005;**65**:1961-72.
4. Lucs AV, Muller WJ, Muthuswamy SK. Shc is required for ErbB2-induced inhibition of apoptosis but is dispensable for cell proliferation and disruption of cell polarity. *Oncogene* 2010;**29**:174-87.
5. Wang J, Yin Y, Hua H, Li M, Luo T, Xu L, Wang R, Liu D, Zhang Y, Jiang Y. Blockade of GRP78 sensitizes breast cancer cells to microtubules-interfering agents that induce the unfolded protein response. *J Cell Mol Med* 2009;**13**:3888-97.
6. Lin MT, Chang CC, Chen ST, Chang HL, Su JL, Chau YP, Kuo ML. Cyr61 expression confers resistance to apoptosis in breast cancer MCF-7 cells by a mechanism of NF-kappaB-dependent XIAP up-regulation. *J Biol Chem* 2004;**279**:24015-23.
7. Yang C, He L, He P, Liu Y, Wang W, He Y, Du Y, Gao F. Increased drug resistance in breast cancer by tumor-associated macrophages through IL-10/STAT3/bcl-2 signaling pathway. *Med Oncol* 2015;**32**:352.
8. Sung M, Giannakakou P. BRCA1 regulates microtubule dynamics and taxane-induced apoptotic cell signaling. *Oncogene* 2014;**33**:1418-28.
9. Lafarge S, Sylvain V, Ferrara M, Bignon YJ. Inhibition of BRCA1 leads to increased chemoresistance to microtubule-interfering agents, an effect that involves the JNK pathway. *Oncogene* 2001;**20**:6597-606.

10. Wertz IE, Kusam S, Lam C, Okamoto T, Sandoval W, Anderson DJ, Helgason E, Ernst JA, Eby M, Liu J, Belmont LD, Kaminker JS, et al. Sensitivity to antitubulin chemotherapeutics is regulated by MCL1 and FBW7. *Nature* 2011;**471**:110-4.
11. Wang H, Liu B, Zhang C, Peng G, Liu M, Li D, Gu F, Chen Q, Dong JT, Fu L, Zhou J. Parkin regulates paclitaxel sensitivity in breast cancer via a microtubule-dependent mechanism. *J Pathol* 2009;**218**:76-85.
12. Sun X, Li D, Yang Y, Ren Y, Li J, Wang Z, Dong B, Liu M, Zhou J. Microtubule-binding protein CLIP-170 is a mediator of paclitaxel sensitivity. *J Pathol* 2012;**226**:666-73.
13. Xu M, Takanashi M, Oikawa K, Nishi H, Isaka K, Yoshimoto T, Ohyashiki J, Kuroda M. Identification of a novel role of Septin 10 in paclitaxel-resistance in cancers through a functional genomics screen. *Cancer Sci* 2012;**103**:821-7.
14. Tan M, Jing T, Lan KH, Neal CL, Li P, Lee S, Fang D, Nagata Y, Liu J, Arlinghaus R, Hung MC, Yu D. Phosphorylation on tyrosine-15 of p34(Cdc2) by ErbB2 inhibits p34(Cdc2) activation and is involved in resistance to taxol-induced apoptosis. *Mol Cell* 2002;**9**:993-1004.
15. Cai J, Chen S, Zhang W, Zheng X, Hu S, Pang C, Lu J, Xing J, Dong Y. Salvianolic acid A reverses paclitaxel resistance in human breast cancer MCF-7 cells via targeting the expression of transgelin 2 and attenuating PI3 K/Akt pathway. *Phytomedicine* 2014;**21**:1725-32.
16. Bin JW, Wong IL, Hu X, Yu ZX, Xing LF, Jiang T, Chow LM, Biao WS. Structure-activity relationship study of permethyl ningalin B analogues as P-glycoprotein chemosensitizers. *J Med Chem* 2013;**56**:9057-70.
17. Li QQ, Cao XX, Xu JD, Chen Q, Wang WJ, Tang F, Chen ZQ, Liu XP, Xu ZD. The role of P-glycoprotein/cellular prion protein interaction in multidrug-resistant breast cancer cells treated with paclitaxel. *Cell Mol Life Sci* 2009;**66**:504-15.
18. Shi JF, Yang N, Ding HJ, Zhang JX, Hu ML, Leng Y, Han X, Sun YJ. ERalpha directly activated the MDR1 transcription to increase paclitaxel-resistance of ERalpha-positive breast cancer cells in vitro and in vivo. *Int J Biochem Cell Biol* 2014;**53**:35-45.
19. Wu CH, Hong BH, Ho CT, Yen GC. Targeting cancer stem cells in breast cancer: the potential anticancer properties of 6-shogaol and pterostilbene. *J Agric Food Chem* 2015;
20. Banerjee A, Qian P, Wu ZS, Ren X, Steiner M, Bougen NM, Liu S, Liu DX, Zhu T, Lobie PE. Artemin stimulates radio- and chemo-resistance by promoting TWIST1-BCL-2-dependent cancer stem cell-like behavior in mammary carcinoma cells. *J Biol Chem* 2012;**287**:42502-15.
21. Ajabnoor GM, Crook T, Coley HM. Paclitaxel resistance is associated with switch from apoptotic to autophagic cell death in MCF-7 breast cancer cells. *Cell Death Dis* 2012;**3**:e260.
