# Supplementary material for: Micro RNA 100 sensitizes luminal A breast cancer cells to paclitaxel treatment in part by targeting mTOR
Source: Oncotarget. 2015 Dec 29;7(5):5702–14. doi: 10.18632/oncotarget.6790 (PMC4868715; doi:10.18632/oncotarget.6790)
Supplement: Supplementary file 3 [file oncotarget-07-5702-s003.pdf]

Table S3. Target molecules of miR-100 as identified in previous studies.

Gene	Function	Organ/tumor
mTOR/AKT		
mTOR [1]	Sensitive to cisplatin	Chondrosarcoma
mTOR [2]	Promoted apoptosis	Esophageal squamous cell carcinoma
mTOR [3]	Inhibited migration and invasion	Esophageal squamous cell carcinoma
mTOR, IGF-1R [4]	Promoted autophagy	Hepatocellular carcinoma
IGF1R [5]	Promoted H2O2 induced apoptosis	Retinal ganglion cells
mTOR [6]	Inhibited carcinogenesis	Bladder
IGF1R, mTOR, AKT1 [7]	Inhibited dermal wound healing (migration)	Skin
mTOR [8]	Inhibited neovascularization	Endothelial and vascular smooth muscle cells
IGF1R, mTOR [9]	Suppressed cell proliferation and migration, and enhanced apoptosis	Head and neck squamous cell carcinoma
mTOR [10]	Reduced tumor cell growth	Childhood adrenocortical tumors
FKBP51, IGF1R/mTOR [11]	Suppressed proliferation and promoted apoptosis	Acute lymphoblastic leukaemia
mTOR [12]	Enhanced sensitivity to the rapamycin analog RAD001	Clear cell ovarian cancer
IGF2 [13]	Inhibits tumorigenesis	Breast cancer
FGFR3 [14]**.**	inhibited proliferation and increased sensitivities to cisplatin	Pancreatic cancer
FGFR3 [15]	Inhibited cell survival	Non-muscle invasive bladder cancer

PLK1		
PLK1	Sensitive to hormone therapy	Basal-like breast cancer
PLK1 [16]	Suppressed carcinogenesis	Hepatocarcinogenesis
PLK1 [17]	Inhibit cell growth	Ovarian cancer
PLK1 [18]	Induced apoptosis and arrested cell cycle.	Non-small cell lung cancer
PLK1 [19]	Sensitive to docetaxel	Lung adenocarcinoma
PLK1 [20]	Inhibited proliferation and migration	Prostate
PLK1 [21]	Inhibited growth and induced apoptosis	Hepatocellular carcinoma
PLK1 [22]	Influenced cell proliferation, cycle and apoptosis	Cervical cancer
PLK1 [23]	Inhibited NPC progression	nasopharyngeal cancer
Others		
SMARCA5, SMARCD1, BMPR2 [25]	Inhibited Self-Renewal of Breast Cancer Stem-like Cells	Breast Cancer
SMARCA5; HOXA1 [26]	Induced EMT but suppressed tumorigenesis, migration and invasion	Breast Cancer
SMARCA5 [27]	Promoted differentiation	Embryonic stem cells
ICMT, Rac1 [28]	Inhibited metastasis	Hepatocellular carcinoma
HoxA1 [29]	Sensitive to Chemotherapy (including ADM, DDP, VP-16)	Small cell lung cancer
RAP1B [30]	Inhibited cell growth and invasion and induced apoptosis	Colorectal cancer
Cyr61 [31]	Inhibited proliferation	Osteosarcoma

SNF2H [32]	Reduced BRCA1 localization to sites of DNA damage	Not specific such as breast cancer
SMRT/NCOR2 [33]	Reduced proliferation, and improved survival	Glioblastoma
RBSP3 [34]	Promoted proliferation, and arrested differentiation	Acute myeloid leukemia
BMPR2 [35]	Inhibited osteogenic differentiation	Human adipose-derived mesenchymal stem cells
beta-tubulin I, IIA, IIB and V [36]	Prevented paclitaxel-induced increases in beta-tubulin isotypes	Breast cancer
ATM [37]	Sensitive to ionizing radiation	Human glioma (M059J)

References:

1. Zhu Z, Wang CP, Zhang YF, Nie L. MicroRNA-100 resensitizes resistant chondrosarcoma cells to cisplatin through direct targeting of mTOR. *Asian Pac J Cancer Prev* 2014;**15**:917-23.
2. Sun J, Chen Z, Tan X, Zhou F, Tan F, Gao Y, Sun N, Xu X, Shao K, He J. MicroRNA-99a/100 promotes apoptosis by targeting mTOR in human esophageal squamous cell carcinoma. *Med Oncol* 2013;**30**:411.
3. Zhang N, Fu H, Song L, Ding Y, Wang X, Zhao C, Zhao Y, Jiao F, Zhao Y. MicroRNA-100 promotes migration and invasion through mammalian target of rapamycin in esophageal squamous cell carcinoma. *Oncol Rep* 2014;**32**:1409-18.
4. Ge YY, Shi Q, Zheng ZY, Gong J, Zeng C, Yang J, Zhuang SM. MicroRNA-100 promotes the autophagy of hepatocellular carcinoma cells by inhibiting the expression of mTOR and IGF-1R. *Oncotarget* 2014;**5**:6218-28.
5. Kong N, Lu X, Li B. Downregulation of microRNA-100 protects apoptosis and promotes neuronal growth in retinal ganglion cells. *BMC Mol Biol* 2014;**15**:25.
6. Xu C, Zeng Q, Xu W, Jiao L, Chen Y, Zhang Z, Wu C, Jin T, Pan A, Wei R, Yang B, Sun Y. miRNA-100 inhibits human bladder urothelial carcinogenesis by directly targeting mTOR. *Mol Cancer Ther* 2013;**12**:207-19.
7. Jin Y, Tymen SD, Chen D, Fang ZJ, Zhao Y, Dragas D, Dai Y, Marucha PT, Zhou X. MicroRNA-99 family targets AKT/mTOR signaling pathway in dermal wound healing. *PLoS One* 2013;**8**:e64434.
8. Grundmann S, Hans FP, Kinniry S, Heinke J, Helbing T, Bluhm F, Sluijter JP, Hoefer I, Pasterkamp G, Bode C, Moser M. MicroRNA-100 regulates neovascularization by suppression of mammalian target of rapamycin in endothelial and vascular smooth muscle cells. *Circulation* 2011;**123**:999-1009.

9. Chen Z, Jin Y, Yu D, Wang A, Mahjabeen I, Wang C, Liu X, Zhou X. Down-regulation of the microRNA-99 family members in head and neck squamous cell carcinoma. *Oral Oncol* 2012;**48**:686-91.
10. Doghman M, El Wakil A, Cardinaud B, Thomas E, Wang J, Zhao W, Peralta-Del Valle MH, Figueiredo BC, Zambetti GP, Lalli E. Regulation of insulin-like growth factor-mammalian target of rapamycin signaling by microRNA in childhood adrenocortical tumors. *Cancer Res* 2010;**70**:4666-75.
11. Li XJ, Luo XQ, Han BW, Duan FT, Wei PP, Chen YQ. MicroRNA-100/99a, deregulated in acute lymphoblastic leukaemia, suppress proliferation and promote apoptosis by regulating the FKBP51 and IGF1R/mTOR signalling pathways. *Br J Cancer* 2013;**109**:2189-98.
12. Nagaraja AK, Creighton CJ, Yu Z, Zhu H, Gunaratne PH, Reid JG, Olokpa E, Itamochi H, Ueno NT, Hawkins SM, Anderson ML, Matzuk MM. A link between mir-100 and FRAP1/mTOR in clear cell ovarian cancer. *Mol Endocrinol* 2010;**24**:447-63.
13. Gebeshuber CA, Martinez J. miR-100 suppresses IGF2 and inhibits breast tumorigenesis by interfering with proliferation and survival signaling. *Oncogene* 2013;**32**:3306-10.
14. Li Z, Li X, Yu C, Wang M, Peng F, Xiao J, Tian R, Jiang J, Sun C. MicroRNA-100 regulates pancreatic cancer cells growth and sensitivity to chemotherapy through targeting FGFR3. *Tumour Biol* 2014;**35**:11751-9.
15. Blick C, Ramachandran A, Wigfield S, McCormick R, Jubb A, Buffa FM, Turley H, Knowles MA, Cranston D, Catto J, Harris AL. Hypoxia regulates FGFR3 expression via HIF-1alpha and miR-100 and contributes to cell survival in non-muscle invasive bladder cancer. *Br J Cancer* 2013;**109**:50-9.
16. Petrelli A, Carollo R, Cargnelutti M, Iovino F, Callari M, Cimino D, Todaro M, Mangiapane LR, Giammona A, Cordova A, Montemurro F, Taverna D, et al. By promoting cell differentiation, miR-100 sensitizes basal-like breast cancer stem cells to hormonal therapy. *Oncotarget* 2014;
17. Petrelli A, Perra A, Schernhuber K, Cargnelutti M, Salvi A, Migliore C, Ghiso E, Benetti A, Barlati S, Ledda-Columbano GM, Portolani N, De Petro G, et al. Sequential analysis of multistage hepatocarcinogenesis reveals that miR-100 and PLK1 dysregulation is an early event maintained along tumor progression. *Oncogene* 2012;**31**:4517-26.
18. Peng DX, Luo M, Qiu LW, He YL, Wang XF. Prognostic implications of microRNA-100 and its functional roles in human epithelial ovarian cancer. *Oncol Rep* 2012;**27**:1238-44.
19. Liu J, Lu KH, Liu ZL, Sun M, De W, Wang ZX. MicroRNA-100 is a potential molecular marker of non-small cell lung cancer and functions as a tumor suppressor by targeting polo-like kinase 1. *BMC Cancer* 2012;**12**:519.
20. Feng B, Wang R, Chen LB. MiR-100 resensitizes docetaxel-resistant human lung adenocarcinoma cells (SPC-A1) to docetaxel by targeting Plk1. *Cancer Lett* 2012;**317**:184-91.
21. Giangreco AA, Vaishnav A, Wagner D, Finelli A, Fleshner N, Van der Kwast T, Vieth R, Nonn L. Tumor suppressor microRNAs, miR-100 and -125b, are regulated by 1,25-dihydroxyvitamin D in primary prostate cells and in patient tissue. *Cancer Prev Res (Phila)* 2013;**6**:483-94.
22. Chen P, Zhao X, Ma L. Downregulation of microRNA-100 correlates with tumor progression and poor prognosis in hepatocellular carcinoma. *Mol Cell Biochem* 2013;**383**:49-58.

23. Li BH, Zhou JS, Ye F, Cheng XD, Zhou CY, Lu WG, Xie X. Reduced miR-100 expression in cervical cancer and precursors and its carcinogenic effect through targeting PLK1 protein. *Eur J Cancer* 2011;**47**:2166-74.
24. Shi W, Alajez NM, Bastianutto C, Hui AB, Mocanu JD, Ito E, Busson P, Lo KW, Ng R, Waldron J, O'Sullivan B, Liu FF. Significance of Plk1 regulation by miR-100 in human nasopharyngeal cancer. *Int J Cancer* 2010;**126**:2036-48.
25. Deng L, Shang L, Bai S, Chen J, He X, Martin-Trevino R, Chen S, Li XY, Meng X, Yu B, Wang X, Liu Y, et al. MicroRNA100 inhibits self-renewal of breast cancer stem-like cells and breast tumor development. *Cancer Res* 2014;**74**:6648-60.
26. Chen D, Sun Y, Yuan Y, Han Z, Zhang P, Zhang J, You MJ, Teruya-Feldstein J, Wang M, Gupta S, Hung MC, Liang H, et al. miR-100 induces epithelial-mesenchymal transition but suppresses tumorigenesis, migration and invasion. *PLoS Genet* 2014;**10**:e1004177.
27. Tarantino C, Paolella G, Cozzuto L, Minopoli G, Pastore L, Parisi S, Russo T. miRNA 34a, 100, and 137 modulate differentiation of mouse embryonic stem cells. *FASEB J* 2010;**24**:3255-63.
28. Zhou HC, Fang JH, Luo X, Zhang L, Yang J, Zhang C, Zhuang SM. Downregulation of microRNA-100 enhances the ICMT-Rac1 signaling and promotes metastasis of hepatocellular carcinoma cells. *Oncotarget* 2014;
29. Xiao F, Bai Y, Chen Z, Li Y, Luo L, Huang J, Yang J, Liao H, Guo L. Downregulation of HOXA1 gene affects small cell lung cancer cell survival and chemoresistance under the regulation of miR-100. *Eur J Cancer* 2014;**50**:1541-54.
30. Peng H, Luo J, Hao H, Hu J, Xie SK, Ren D, Rao B. MicroRNA-100 regulates SW620 colorectal cancer cell proliferation and invasion by targeting RAP1B. *Oncol Rep* 2014;**31**:2055-62.
31. Huang J, Gao K, Lin J, Wang Q. MicroRNA-100 inhibits osteosarcoma cell proliferation by targeting Cyr61. *Tumour Biol* 2014;**35**:1095-100.
32. Mueller AC, Sun D, Dutta A. The miR-99 family regulates the DNA damage response through its target SNF2H. *Oncogene* 2013;**32**:1164-72.
33. Alrfaei BM, Vemuganti R, Kuo JS. microRNA-100 targets SMRT/NCOR2, reduces proliferation, and improves survival in glioblastoma animal models. *PLoS One* 2013;**8**:e80865.
34. Zheng YS, Zhang H, Zhang XJ, Feng DD, Luo XQ, Zeng CW, Lin KY, Zhou H, Qu LH, Zhang P, Chen YQ. MiR-100 regulates cell differentiation and survival by targeting RBSP3, a phosphatase-like tumor suppressor in acute myeloid leukemia. *Oncogene* 2012;**31**:80-92.
35. Zeng Y, Qu X, Li H, Huang S, Wang S, Xu Q, Lin R, Han Q, Li J, Zhao RC. MicroRNA-100 regulates osteogenic differentiation of human adipose-derived mesenchymal stem cells by targeting BMPR2. *FEBS Lett* 2012;**586**:2375-81.
36. Lobert S, Jefferson B, Morris K. Regulation of beta-tubulin isotypes by micro-RNA 100 in MCF7 breast cancer cells. *Cytoskeleton (Hoboken)* 2011;**68**:355-62.
37. Ng WL, Yan D, Zhang X, Mo YY, Wang Y. Over-expression of miR-100 is responsible for the low-expression of ATM in the human glioma cell line: M059J. *DNA Repair (Amst)* 2010;**9**:1170-5.
